# Methods to Analyze the Non-Coding RNA Interactome—Recent Advances and Challenges

**DOI:** 10.3389/fgene.2022.857759

**Published:** 2022-03-17

**Authors:** Huifen Cao, Philipp Kapranov

**Affiliations:** Institute of Genomics, School of Medicine, Huaqiao University, Xiamen, China

**Keywords:** RNA interactome, non-coding RNAs, long non-coding RNAs, RNA dark matter, functional genomics

## Abstract

Most of the human genome is transcribed to generate a multitude of non-coding RNAs. However, while these transcripts have generated an immense amount of scientific interest, their biological function remains a subject of an intense debate. Understanding mechanisms of action of non-coding RNAs is a key to addressing the issue of biological relevance of these transcripts. Based on some well-understood non-coding RNAs that function inside the cell by interacting with other molecules, it is generally believed many other non-coding transcripts could also function in a similar fashion. Therefore, development of methods that can map RNA interactome is the key to understanding functionality of the extensive cellular non-coding transcriptome. Here, we review the vast progress that has been made in the past decade in technologies that can map RNA interactions with different sites in DNA, proteins or other RNA molecules; the general approaches used to validate the existence of novel interactions; and the challenges posed by interpreting the data obtained using the interactome mapping methods.

## Introduction

Even though most (97–98%) of the human genome sequence does not encode exons of protein-coding genes, most of it is transcribed to generate a plethora of apparently non-coding long and short RNAs in a phenomenon referred to as “pervasive transcription” (Kapranov et al., 2002; Okazaki et al., 2002). In fact, the ENCODE consortium estimated that as much as 75% of the human genome is used to encode RNAs, most of which do not have obvious protein-coding potential (Bernstein et al., 2012; Djebali et al., 2012). The original discovery of the pervasive transcription is consistent with the hypothesis that postulates presence of a hidden layer of RNA-based regulation in complex organisms (Mattick, 1994, 2003, 2007; St Laurent and Wahlestedt, 2007) and as such, created significant interest in the non-coding RNA products of the pervasive transcription (Clark et al., 2013; Yang et al., 2014), sometimes collectively referred to as “RNA dark matter” (Kapranov and St Laurent, 2012). However, while the existence of the dark matter transcripts is now well established, their biological relevance has been and still is a subject of debate (Struhl, 2007; Eddy, 2012; Doolittle, 2013; Niu and Jiang, 2013; Palazzo and Gregory, 2014; Raabe and Brosius, 2015). Arguably, the main reasons behind the skepticism are the general paucity of clear phenotypes, with exception of specific examples, in animals (especially vertebrates) that could be unambiguously associated with the dark matter RNAs (Gao et al., 2020), and lack of clear understanding of the mechanisms of function of these transcripts (Mudge et al., 2013).

Nonetheless, the past decade has seen remarkable progress in understanding molecular mechanisms of functions of non-coding RNAs, particularly in the area of mapping intermolecular interactions between these transcripts and other molecules. Uncovering such interactions could likely hold the key to figuring out the mechanisms of function of non-coding transcripts and potentially their biological relevance. The conceptual foundation of this assumption is, at least in a large part, rooted in the pioneering work of multiple groups that studied mechanisms of dosage compensation of genes located on sex chromosomes. Animals, where females have two X chromosomes and males have only one, change expression levels of most of X-linked genes to achieve gene dosage parity between the two genders (Meller, 2000; Payer and Lee, 2008). Non-coding RNAs are the key functional components of the cellular machineries that make it happen in different species, with the *Drosophila* dosage compensation system being one of the best understood from both biochemical and genetic perspectives.

*Drosophila* males upregulate the X-linked genes via action of Male-Specific Lethal (MSL) complex that binds to hundreds of specific, well-characterized sites on the X chromosome (Alekseyenko et al., 2006; Alekseyenko et al., 2008) and changes the chromatin environment at least in part by acetylation of histone H4 at lysine 16 leading to the ∼2 fold induction of gene expression (Akhtar and Becker, 2000; Smith et al., 2000). Besides the protein components, the complex also contains two long non-coding (lnc) RNA transcripts of about 3.7 and 0.6 kb encoded by respectively *roX1* and *roX2* genes. Taken together, several independent lines of evidence have conclusively proven that the *roX* transcripts target the MSL complex to the specific sites on the X chromosome and represent critical components of the dosage compensation machinery. First, the *roX* transcripts have the same localization pattern on the X chromosome as the MSL complex (Meller et al., 1997; Franke and Baker, 1999). Second, binding of the MSL complex to the X chromosome is sensitive to RNase (Richter et al., 1996; Akhtar et al., 2000). Third, the *roX* transcripts form stable association with the protein components of the complex (Akhtar et al., 2000; Gu et al., 2000; Meller et al., 2000). Fourth, an elegant recent study has shown that ectopic dosage compensation could be induced in a heterologous mammalian system that expresses only *roX2* lncRNA and the mammalian MSL2 protein containing the C-terminal domain (CTD) of the *Drosophila* MSL2 (Valsecchi et al., 2021). Strikingly, interaction between *roX2* and MSL2 CTD changed the biophysical properties of the latter leading to formation of a stably condensed state that the authors suggest is critical for the dosage compensation mechanism (Valsecchi et al., 2021).

The final evidence comes from the genetic studies that showed that while the two *roX* genes are redundant, combined knockout of both genes leads to male-specific reduction in viability (Meller and Rattner, 2002) and loss of MSL complex localization to the X chromosome (Franke and Baker, 1999). Importantly, the phenotype can be rescued by ectopic expression of *roX* cDNAs, encoded on the X chromosome in the wild type flies, from transgenes integrated in autosomes, thus unambiguously proving the functional relevance of these transcripts (Meller and Rattner, 2002). In fact, the *roX* transcripts represent example of few lncRNAs for which phenotypes in animals have been unambiguously connected to the corresponding transcripts *via* the rescue confirmation experiments (Gao et al., 2020). And, the *roX* transcripts also illustrate the importance of the phenotype rescue since both *roX* RNAs also overlap DNA binding sites for the MSL complex. Therefore, without the rescue confirmation, a possibility would have existed that deletions of both transcripts exerted their phenotypes not *via* depletion of the transcripts, but by abrogation of the MSL entry sites (Meller and Rattner, 2002).

RNA-mediated targeting is also the key component in eutherian dosage compensation mechanism that results in inactivation of most of genes on one out of the two X-chromosomes in females. A long (∼17 kb in human and ∼15 kb in mouse) spliced non-coding RNA *XIST* is transcribed from a specific location (X-inactivation center or XIC) on the X-chromosome to be inactivated (Brown et al., 1991) and remains associated with the inactive X-chromosome (Clemson et al., 1996) leading to creation of a transcriptionally-repressive nuclear compartment (Chaumeil et al., 2006) *via* targeting of the PRC2 Polycomb complex to the inactivated X chromosome (Zhao et al., 2008) [reviewed in (Lee, 2011)].

**FIGURE 1:**
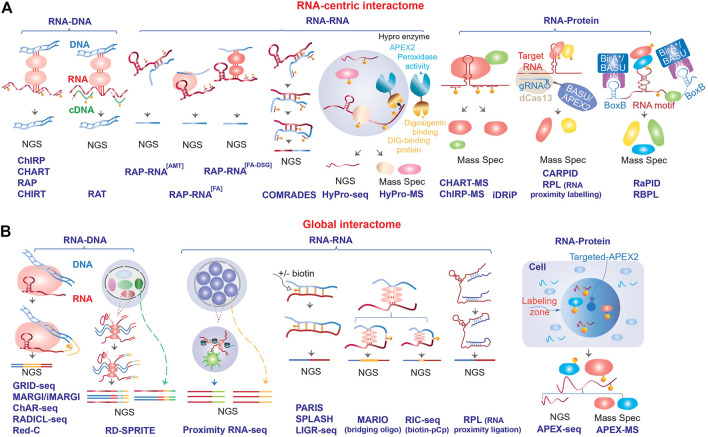
Schematic outline of the different methods used to map RNA interactome. The methods are divided into **(A)** RNA-centric and **(B)** global, and further stratified based on the type of interactions (RNA-DNA, RNA-RNA or RNA-protein) that they are designed to map (see Table 1 for more details). NGS, next generation sequencing.

Altogether, the dosage compensation lncRNAs provided a paradigm of how at least a fraction of the dark matter RNAs might function: targeting of specific proteins or protein complexes, such as chromatin modifiers for example, to specific locations in the genome and modulating gene expression by creating subcellular compartments and/or changing local chromatin environment (Kung et al., 2013; Mercer and Mattick, 2013; Bergmann and Spector, 2014). Combined with the observations that the RNA products of the pervasive non-coding transcription tend to be enriched in nucleus (Cheng et al., 2005; Kapranov et al., 2007), this mechanism of action becomes an attractive potential mechanism of function for a large fraction of the dark matter RNAs. Therefore, identification of the binding partners of lncRNAs—the interactomes of these transcripts—is a critical step towards elucidation of the mechanisms of action of this class of transcripts and the ability to perform reliable measurements of these interactions is the foundation of this endeavor. Below, we review recent progress in techniques and approaches to map RNA interactome and highlight a number of questions and challenges that were posed by these studies. While the focus of this review is on the non-coding RNA interactome, these methods can and have been used to map interactions that involve protein-coding mRNAs since RNA-RNA and RNA-protein interactions are well-known to be critical for regulation of expression of this class of transcripts. In this review, we will focus on two classes of methods used to map RNA interactome that are focused on either analysis of interactomes of a specific transcript or RNA motif (RNA-centric, Figure 1A and Table 1) or mapping global interactions involving all RNA molecules (Figure 1B; Table 1).

**TABLE 1:** Summary of the RNA interactome mapping methods.

Type	Method name	Interaction detected	Crosslinkers^a^ used	Estimated distance of measured interactions^b^	Basic principle	Level of relative technical and analytical complexity^c^	Reference
RNA-centric	ChIRP	RNA-DNA	GA or FA	Non-proximal	Affinity purification of fragmented chromatin using affinity tagged oligonucleotides against an RNA of interest	I	Chu et al. (2011)
	CHART		FA				Simon et al. (2011)
	RAP		DSG + FA				Engreitz et al. (2013)
	CHIRT		GA				Chu et al. (2017)
	RAT		FA		*In situ* cDNA synthesis primed by oligonucleotides against an RNA of interest in presence of biotinylated deoxynucleotides followed by chromatin fragmentation and affinity purification		Sun et al. (2014)
	COMRADES	RNA-RNA	PS-based	Direct base pairing	Affinity purification of crosslinked RNA molecules using affinity tagged oligonucleotides (one or many) against an RNA of interest		Ziv et al. (2018)
	RAP-RNA		PS-based (RAP-RNA^[AMT]^)				Engreitz et al. (2014)
			FA (RAP-RNA^[FA]^)	Non-proximal	Affinity purification of fragmented chromatin using affinity tagged oligonucleotides against an RNA of interest		
			DSG + FA (RAP-RNA^[FA−DSG]^)				
	HyPro-seq						
	HyPro-MS	RNA-protein	DSP	Within ∼20 nm	Proximal biotinylation by APEX2 targeted to an RNA of interest using affinity tagged oligonucleotides in crosslinked and permeabilized cells	II	Yap et al. (2021)
	CHART-MS		FA	Non-proximal	Affinity purification of fragmented chromatin using affinity tagged oligonucleotides against an RNA of interest		West et al. (2014)
	ChIRP-MS		FA				Chu et al. (2015)
	iDRiP		UV	Direct binding			Minajigi et al. (2015), Chu et al. (2021)
	CARPID		None	Within ∼25 nm of target RNA	Proximal biotinylation by APEX2 or BASU targeted to an RNA of interest using CRISPR/dCas13 *in vivo*		Yi et al. (2020)
	RPL (RNA proximity labelling)						Lin et al. (2021)
	RaPID				Proximal biotinylation by BirA^a^ or BASU targeted to an RNA motif of interest using a two component RNA/protein system *in vivo*		Ramanathan et al. (2018)
	RBPL						Lu and Wei, (2019)
Global	GRID-seq	RNA-DNA	DSG + FA	Proximal	Proximity ligation mediated by affinity-tagged bridge oligonucleotides	III	Li et al. (2017)
	MARGI/iMARGI		FA, DSG + FA				Sridhar et al. (2017), Yan et al. (2019)
	ChAR-seq		FA				Bell et al. (2018)
	RADICL-seq		FA				Bonetti et al. (2020)
	Red-C		FA				Razin et al. (2020)
	RD-SPRITE	RNA-RNA, RNA-DNA, DNA-DNA	DSG + FA	Non-proximal	Adding the same barcode on all RNA or all RNA and DNA molecules within the same subnuclear particle obtained after chromatin fragmentation	IV	Quinodoz et al. (2021)
	Proximity RNA-seq	RNA-RNA	EGS + FA				Morf et al. (2019)
	PARIS		PS-based	Direct base pairing	Direct proximity ligation	III	Lu et al. (2016)
	SPLASH						Aw et al. (2016)
	LIGR-seq						Sharma et al. (2016)
	MARIO		UV	Proximal	Proximity ligation mediated by affinity-tagged bridge oligonucleotides		Nguyen et al. (2016)
			EGS + FA				
	RIC-seq		FA		Proximity ligation mediated by affinity-tagged small molecule		Cai et al. (2020)
	RPL (RNA proximity ligation)		None		Direct proximity ligation		Ramani et al. (2015)
	APEX-seq	RNA-protein		Variable <100 nm	Targeting of APEX2 to a specific subcellular locale *in vivo* followed by correlation of results obtained using both methods		Fazal et al. (2019), Padron et al. (2019)
	APEX-MS						Padron et al. (2019)

a Formaldehyde (FA); glutaraldehyde (GA); UV light (UV); different psoralen-based compounds (PS-based); disuccinimidyl glutarate (DSG); ethylene glycol-bis(succinimidylsuccinate) (ESG); dithio-bis(succinimidyl propionate) (DSP).

b Proximal interactions would include detection of events where molecules are directly bound to each other as well as nearby indirect, protein-mediated, interactions. Non-proximal would include direct, and also both nearby and distal indirect interactions (see text for more details).

c Relative complexity based on wet lab and analytical components of the procedure, and estimated time and cost of the protocol, with the level I being the easiest.

## RNA-centric Interactome Analysis Methods

The first techniques to analyze interactome of a specific transcript focused on RNA-DNA interactions and were similar in many ways to the widely used ChIP-seq (chromatin immunoprecipitation followed by sequencing) suite of methods where protein-DNA interactions are mapped genome-wide using immunoprecipitation based on *in vivo* cross-linked (to preserve *in vivo* interactions, see below) and fragmented chromatin with an antibody to a protein of interest. The major difference is that instead of an antibody, several pioneering RNA centric interactome mapping techniques such as ChIRP [chromatin isolation by RNA purification, (Chu et al., 2011)], CHART [capture hybridization analysis of RNA targets, (Simon et al., 2011)], RAP [RNA antisense purification, (Engreitz et al., 2013)] and CHIRT (Chu et al., 2017) relied on affinity-tagged oligonucleotides complementary to an RNA of interest to isolate chromatin fraction containing that RNA (Figure 1A; Table 1). On the other hand, RAT (reverse transcription-associated trap) assay provided an interesting variation on the oligonucleotide-mediated chromatin enrichment strategy, where instead of directly purifying RNA-containing complexes, unlabeled oligonucleotides against an lncRNA of interest served as primers for *in situ* cDNA synthesis (using the lncRNA as the template) in cross-linked nuclei in presence of biotinylated deoxynucleotides, followed by streptavidin affinity purification of the chromatin complexes containing the cDNAs [Figure 1A; Table 1, (Sun et al., 2014)].

The original techniques that used the oligonucleotide-based enrichment strategy focused on identification of DNA regions that interacted with different lncRNAs of interest. However, later this strategy was also adapted to identify protein [CHART-MS, (West et al., 2014); ChIRP-MS, (Chu et al., 2015)] or RNA [RAP-RNA, (Engreitz et al., 2014)] interacting partners of specific lncRNAs (Figure 1A; Table 1). The latter study has also shown that different choice of crosslinking reagents can detect either direct interaction (mediated by base-pairing between different RNA molecules), or direct and indirect interactions mediated by proteins bridging different RNA molecules (Figure 1A; Table 1). The commonly used formaldehyde can reversibly crosslink proteins to proteins or proteins to nucleic acids (Hoffman et al., 2015), thus allowing for mapping either direct or indirect interactions. Additional treatment with potent protein-protein crosslinkers, such as disuccinimidyl glutarate (DSG) or ethylene glycol-bis(succinimidylsuccinate) (ESG), that can further stabilize nucleic acid interactions mediated by multiple proteins (Tian et al., 2012), is also used in some RNA-RNA mapping methodologies if a broader view of indirect RNA interactome is desired (Engreitz et al., 2014). UV light at certain wavelength can crosslink nucleic acids to proteins, but not proteins to proteins (Pashev et al., 1991), therefore this crosslinking approach would limit the scope of protein-mediated RNA-RNA interactomes and also limit RNA-protein interactomes to direct interactions (Figure 1A; Table 1). Usage of this crosslinking reagent is a unique feature of iDRiP (identification of direct RNA interacting proteins) methodology designed to identify proteins directly interacting with a specific RNA species (Figure 1A; Table 1) (Minajigi et al., 2015; Chu et al., 2021).

On the other hand, usage of psoralen-based crosslinkers can create reversible interstrand crosslinks in nucleic acid helices (Cimino et al., 1985), thus allowing for exclusive stabilization of direct interactions mediated by regions of base pairing. This class of crosslinkers has been used extensively to map RNA-RNA interactomes in both RNA-centric (Engreitz et al., 2014; Ziv et al., 2018) and global contexts (see below). For example, COMRADES (crosslinking of matched RNAs and deep sequencing) method has combined the oligonucleotide enrichment, psoralen crosslinking and proximal ligation strategies (see below) to identify cellular transcripts interacting with Zika virus RNA genome [(Ziv et al., 2018), Figure 1A and Table 1]. Interestingly, this method relies on an azide-modified crosslinker that can be used to select the crosslinked products thus increasing the efficiency of interactome mapping (Ziv et al., 2018).

A number of more recent RNA-centric techniques are built on a promising proximity labeling technology based on the ability of a peroxidase to generate biotin-phenoxyl radicals in presence of biotin-phenol and hydrogen peroxide (Figure 1A; Table 1). The radicals can then react with nearby protein or RNA molecules in crosslinked or living cells resulting in addition of biotin tags that could be later used for the affinity purification (Figure 1A). When targeted to specific transcripts, the engineered version of the peroxidase APEX (Martell et al., 2012) or APEX2 (Lam et al., 2015) can biotinylate proteins in the immediate vicinity of the targeted transcripts due to the very short half-live of the biotin-phenoxyl radicals (Rhee et al., 2013). In addition to biotinylation of proteins, APEX2 peroxidase can also biotinylate RNA (Fazal et al., 2019; Padron et al., 2019). The peroxidase could be targeted to specific RNAs using antisense oligonucleotides or guide RNAs in the CRISPR/Cas13 system (Figure 1A; Table 1, see below). The HyPro (hybridization-proximity, Figure 1A and Table 1) suite of methods (Yap et al., 2021) is based on the initial targeting of specific transcripts in fixed permeabilized cells with antisense oligonucleotides labelled with digoxigenin. This step is then followed by the addition of a fusion protein containing DIG10.3 digoxigenin-binding domain fused to APEX2, and the APEX2 substrates (Yap et al., 2021). Then, the interacting proteins or RNAs could be profiled using HyPro-MS and HyPro-seq techniques (Yap et al., 2021).

Importantly, the targeting of peroxidase to specific transcripts could also be performed *in vivo* by creating peroxidase fusions with catalytically dead (d) Cas13 enzymes and transfecting constructs encoding the fusion and the targeting guide RNAs into live cells (Figure 1A; Table 1) (Han et al., 2020; Yi et al., 2020; Lin et al., 2021). Therefore, unlike the technologies that require cross-linked nuclei, methods based on proximity labeling can detect *in vivo* interaction without potential artifacts of crosslinking. The proximity labeling methods can also be adapted to study a group of transcripts that share a specific motif, as exemplified by RaPID (RNA–protein interaction detection, Figure 1A and Table 1) methodology based on a modified version of a different type of enzyme that can biotinylate proximal proteins—promiscuous biotin ligase BirA* (Ramanathan et al., 2018). In this study, the authors investigated proteins binding to a specific RNA motif. The application depends on co-expressing two exogenous elements: the RNA component containing an RNA motif of interest fused to an RNA binding site for a 22-amino-acid λN peptide which is recognized by the second component, a protein fusion of the λN peptide fused and the BirA* biotin ligase (Ramanathan et al., 2018). The latter can biotinylate the proteins bound to the RNA motif of interest that could then be affinity purified and analyzed using proteomics methods (Ramanathan et al., 2018). Furthermore, that study also developed BASU, a new mutant version of BirA* with higher ligation efficiency (Ramanathan et al., 2018). BASU was later employed in the RBPL (RNA-bound protein proximity labeling) method, an approach similar to RaPID (Figure 1A; Table 1), but developed to be used in the context of cell lines stably expressing the RNA and protein components (Lu and Wei, 2019).

## Global RNA Interactome Analysis Methods

One of the most popular strategies behind the current global interaction mapping techniques is proximity ligation that allows to map interactions between proximal RNA and DNA or RNA molecules. Similar to RAP, ChART, ChIRP and RAT, these methods also start with cross-linked cells or nuclei to preserve native, *in vivo* interactions. The key feature of the proximity ligation methods that map RNA-DNA interactions—GRID-seq [global RNA interactions with DNA by deep sequencing, (Li et al., 2017)], MARGI [mapping RNA-genome interactions, (Sridhar et al., 2017)] and an enhanced version of MARGI technique developed by the same group [iMARGI, (Yan et al., 2019)], ChAR-seq [chromatin-associated RNA sequencing, (Bell et al., 2018)], RADICL-seq [RNA and DNA interacting complexes ligated and sequenced, (Bonetti et al., 2020)] and Red-C [RNA ends on DNA capture, (Razin et al., 2020)]—is a two-step ligation procedure performed on crosslinked and fragmented chromatin and mediated by a partially double-stranded bridge oligonucleotide (Figure 1B; Table 1). The latter is designed such that, typically, 5′ end is single-stranded and capable of ligation only to a 3′-OH terminus of an RNA molecule in the first ligation step, while the other end of the oligo is double-stranded and capable of subsequent ligation to genomic DNA that has been properly fragmented to ensure compatibility with the oligo (Figure 1B; Table 1). An important additional component of the bridge oligo is the presence of an affinity tag (usually biotin) that allows for affinity selection of the ligation products that could then be subjected to analysis by next generation sequencing (Figure 1B; Table 1). The DNA fragmentation is usually achieved either by digesting chromatin with frequently cutting restriction enzymes (Li et al., 2017; Sridhar et al., 2017; Bell et al., 2018; Razin et al., 2020) or partial digestion with DNase I followed by end-repair (Bonetti et al., 2020). A unique feature of RADICL-seq is RNA fragmentation using RNase H that removed ribosomal RNAs and nascent RNAs bound to the template DNA, thus increasing the fraction of longer range interactions (Bonetti et al., 2020), while the other methodologies do not incorporate specific RNA fragmentations steps, thus relying on 3′OH termini obtained by random RNA fragmentation during the procedure and prior to the ligation step.

While conceptually similar, global methods based on proximity ligation to detect RNA-RNA interactome differ from the RNA-DNA detection methods in two key ways. First, using different crosslinkers that can detect either direct, or both direct and indirect RNA-RNA interactions as mentioned above while the above-mentioned methods that detect RNA-DNA interactions are only focused on the latter. Three global RNA-RNA interactome mapping methods—PARIS [psoralen analysis of RNA interactions and structures, (Lu et al., 2016)], SPLASH [psoralen crosslinked, ligated, and selected hybrids, (Aw et al., 2016)] and LIGR-seq [ligation of interacting RNA followed by high-throughput sequencing, (Sharma et al., 2016)]—used psoralen-derived cross-linkers and thus can detect predominantly base-paired RNA-RNA interactions (Figure 1B; Table 1). Interestingly, SPLASH uses biotinylated crosslinker to allow for affinity selection of cross-linked nucleic acid molecules (Aw et al., 2016). On the other hand, MARIO [mapping RNA interactome *in vivo*, (Nguyen et al., 2016)] used UV- or formaldehyde-based crosslinking, and RIC-seq [RNA *in situ* conformation sequencing, (Cai et al., 2020)] used formaldehyde-based crosslinking that expand the scope of interactomes detected by those methods (Figure 1B; Table 1). It is worth mentioning that while crosslinking is used in most methods based on proximity ligation, this approach has also been successfully tried in native, non-crosslinked cells yeast and human cells in the context of RPL (RNA Proximity Ligation) method to study RNA structure [(Ramani et al., 2015), Figure 1B and Table 1].

Second, the RNA-RNA interactome methods, as expected, use different ligation procedures from the RNA-DNA methods. The three methods that use psoralen-based crosslinkers also rely on a simple, direct, one-step ligation of adjacent RNA ends without bridging oligonucleotides (Aw et al., 2016; Lu et al., 2016; Sharma et al., 2016) (Figure 1B; Table 1). MARIO employs a single-stranded RNA bridge oligo that contains biotin to allow for affinity selection of the ligation products [(Nguyen et al., 2016), Figure 1B and Table 1]. The RIC-seq developers, on the other hand, devised an interesting two-step ligation scheme mediated by a small molecule a biotinylated cytidine (bis) phosphate (pCp–biotin), that allows for highly efficient selection—estimated at ∼90%—of the ligated products [(Cai et al., 2020), Figure 1B and Table 1].

The global methodologies described above predominantly focus on relatively proximal interactions: even when no direct interactions between RNA and its partners are required, proximal ligation would likely favor molecules in close proximity to each other (Quinodoz et al., 2018). Therefore, such methods might be limited in uncovering more distal interactions (Quinodoz et al., 2018), that could still be important for lncRNA functioning, for example organizing the 3D structure of the nucleus. This problem has been creatively solved by the Proximity RNA-seq (Morf et al., 2019) and RD-SPRITE (Quinodoz et al., 2021) methodologies that have addressed this limitation by breaking crosslinked nuclei into multiple particles by sonication and adding the same unique barcodes on all transcripts (Proximity RNA-seq) or all RNA and DNA molecules (RD-SPRITE) within the same particle (Figure 1B; Table 1). Presence of the same barcode on reads derived from different RNA or DNA molecules thus signifies their relative proximity within the nuclear 3D space (Morf et al., 2019; Quinodoz et al., 2021). In Proximity RNA-seq, the particle-specific barcoding step is performed by encapsulating each subnuclear particle in a separate emulsion droplet and performing the reverse transcription and PCR steps in the same droplet (Morf et al., 2019). In RD-SPRITE, this was achieved by a series of successive ligations and dilution steps (Quinodoz et al., 2021). Importantly, since in RD-SPRITE method the barcodes are added to both RNA and DNA molecules in the same particle, this technique can measure proximity of RNA-RNA, RNA-DNA and DNA-DNA molecules (Quinodoz et al., 2021).

The ability of APEX2 to biotinylate both RNA and protein became the basis of APEX-seq and APEX-MS methodologies (Figure 1B; Table 1) that allow for *in vivo* analysis of spatial distribution of RNA and protein populations respectively in very specific subcellular locales by expressing APEX2 targeted to these locations (Fazal et al., 2019; Padron et al., 2019). Correlating spatial RNA and protein localization data derived from these techniques could be used to obtain information on global proximity of different RNA and protein molecules (Padron et al., 2019).

## Advantages and Disadvantages of Different Methods and Strategies to Overcome Them

RNA-centric methods have one clear advantage over the global methods: sensitivity of detection of interactions for a specific RNA of interest (Table 2). This advantage is specifically important for low abundant RNA species—a common feature of most lncRNAs [reviewed in (Cao et al., 2018)]. Moreover, such methods are relatively technically simple as compared to the global methods and have many wet lab and analytical steps that are similar to the commonly used ChIP-seq suite of procedures (Table 1). However, many RNA-centric methods are based on oligonucleotide-mediated enrichment of transcripts of interests (and their interacting partners), and thus the well-known potential for non-specific cross-hybridization of the oligonucleotides to non-targeted locations in the genome is also a major disadvantage of such techniques (Table 2). Therefore, the RNA-centric interactome mapping methods employ a variety of steps in terms of both the design of the assays and controls to ensure the specificity of the detected interactions. Such approaches typically start with careful selection of the probes to avoid sequences that are repetitive in the genome (Chu et al., 2011; Cao et al., 2021; Chu et al., 2021). Furthermore, in some studies, the oligonucleotides targeting the same RNA are split into two non-overlapping pools and the RNA-interactome mapping is performed independently using each pool, and subsequently, only interactions detected using both pools are kept (Chu et al., 2011; Cao et al., 2021).

**TABLE 2:** Advantages and disadvantages of different properties of RNA interactome mapping methods.

Property of an assay	Advantages	Disadvantages
RNA-centric	1. High sensitivity of interactome mapping for a specific transcript of interest	1. Low throughput
	2. Relatively technically and analytically simple	2. High potential for detecting non-specific interactions for the methods based on the oligonucleotide enrichment
Global	Provide system-level view of RNA interactome	1. Technically and analytically complex
		2. Low sensitivity for a specific RNA of interest—a major concern for low abundant RNA species
Oligonucleotide-based enrichment	1. Technically simple	Relatively high propensity for non-specific cross-hybridization
	2. Can be performed on any cell type	
CRISPR/dCas13-based enrichment	1. Higher specificity	The *in vivo* applications are mostly limited to cultured cells
	2. Can be performed *in vivo*	
*In vitro*	Not limited to a particular cell type	May not fully represent the *in vivo* situation
*In vivo*	Represent interactions happening in living cells	Mostly limited to cultured cells
Proximity ligation	Provides sequence information on both interacting partners thus significantly reducing the non-specific noise	1. Technically and analytically complex
		2. Limited to nearby interactions
		3. Exact proximity range is not known
Analysis of crosslinked complexes or subnuclear particles*	1. Not limited to nearby interactions	1. Technically and analytically very complex
	2. Can provide simultaneous information on proximity of RNA-RNA, RNA-DNA and DNA-DNA molecules	2. Exact proximity range is not known
	3. Provides sequence information on both interacting partners thus significantly reducing the non-specific noise	
Proximity labeling	1. Has known proximity range	The *in vivo* applications are mostly limited to cultured cells
	2. Compatible with both *in vitro* and *in vivo* systems	
	3. Can be used to analyze RNA-RNA and RNA-protein interactions	

*Refers to RD-SPRITE, and Proximity RNA-seq, methodologies.

Specificity considerations also result in differences in the number and length of oligonucleotides used against a specific target. Smaller number of oligonucleotides and their shorter lengths should theoretically increase specificity, but could decrease sensitivity. For example, the developers of iDRiP suggested using short (20–25 bases) oligonucleotides with the melting temperatures in the 55–60°C range and sparsely spaced (every 500 nt) in the target RNA (Chu et al., 2021). On the other hand, the RAP methodology utilized high density coverage of the target RNA with overlapping and very long (120 bases) oligonucleotides that allow for purification under high-stringency conditions to ensure specificity (Engreitz et al., 2013). The authors also claim that such designs are required for reliable purification of the target RNA since some parts of the sequence may not be available for binding due to RNA secondary structure or interactions with other molecules (Engreitz et al., 2013).

The COMRADES methodology (Table 1) that combines oligonucleotide-mediated enrichment with psoralen-based crosslinking and proximity ligation would yield sequence information on both interacting partners, thus significantly decreasing the non-specific noise (Ziv et al., 2018). However, this method is so far limited to direct base pairing interactions (Ziv et al., 2018). Specificity of RNA-centric methods can be further improved by using the CRISPR/dCas13 system that is known to have a relatively high specificity (Abudayyeh et al., 2017; Konermann et al., 2018) to target specific RNAs (Table 1). However, such methods also use multiple independent gRNAs targeting the same RNA to ensure specificity (Lin et al., 2021).

On the other hand, the global methods can provide a very broad view of RNA-interactome highly desirable for the systems-based studies, albeit with likely reduced sensitivity for specific transcripts (Table 2). However, such methods are significantly more complex, both in terms of the wet lab procedures and the analytical components (Tables 1 and 2). For example, the methods that depend on proximity ligation (Table 1) face immediate challenge of accurately mapping the chimeric sequencing reads, containing sequences derived from two different molecules, to the genome. This problem is somewhat exacerbated by the fact that some of these techniques generate only short (as short as 20 bases) sequence tag representing each or both interacting partners [as in the case of GRID-seq (Li et al., 2017)], and thus presenting a challenge of accurately mapping such short sequences to complex genomes. Therefore, to overcome this problem, the RADICL-seq technique increased the length of tags of both the DNA and RNA partners to 27 bases that significantly improved the accuracy of the mapping (Bonetti et al., 2020). On the other hand, the Red-C methodology, while limited to 20 base-long tags representing the DNA partners, can potentially obtain sequence of the entire RNA molecules associated with the DNA sequence, thus significantly simplifying the task of precisely locating the RNA partners in the genome (Razin et al., 2020). Furthermore, the knowledge of the extended sequence of the RNA partner can be very helpful in assigning interactomes to specific transcript isoforms.

However, the additional bioinformatic challenges are not limited to mapping of the chimeric reads and extend to all downstream aspects of the analysis since these global methods generate information whose structure is intrinsically much more complex compared to that of the RNA-centric methods. Therefore, to address the issues of dealing with mapping of the chimeric reads and other downstream analytical and interpretational challenges, some of the groups that develop global methodologies also make publicly available corresponding suites of bioinformatics methods that would allow users to analyze their own data, such as, for example, MARIO tools for the analysis and visualization of the MARIO data (Nguyen et al., 2016).

Considering the complexities of the methodologies and potential for detection of non-specific interactions, in addition to the experimental design, a number of common controls are often incorporated into the RNA interactome mapping studies. As expected, the RNA-centric methods (Table 1) often include experiments to control for oligonucleotide specificity, for example, performed with oligonucleotides in the sense polarity of the targeting RNAs (and thus not expected to bind to these transcripts) and/or scrambled sequences to estimate contribution of the genomic DNA and non-specific binding to the resulting signal [for example, (Simon et al., 2011; Engreitz et al., 2013; West et al., 2014; Chu et al., 2021; Yap et al., 2021)]; or performed in the absence of the targeting oligonucleotides to estimate the contribution of experimental noise (Cao et al., 2021; Yap et al., 2021). Similarly, the *in vivo* RNA-interactome mapping techniques that rely on CRISPR/dCas13 (Table 1) employ non-specific gRNA or empty vectors that do not express gRNAs as controls (Yi et al., 2020; Lin et al., 2021).

The techniques that map global RNA-DNA or RNA-RNA interactions based on proximity ligation (Table 1) often incorporate parallel experiments that omit the ligation step to control for the specificity of the assays (Sharma et al., 2016; Sridhar et al., 2017; Razin et al., 2020). An additional common control strategy is to mix cell lysates from distant species prior to the assays and then use the fraction of inter-species chimeric reads to estimate the fraction of non-specific interactions detected by the assays [for example, (Nguyen et al., 2016; Sridhar et al., 2017)]. Various RNA-centric and global methodologies also often include controls where RNA is destroyed by RNase treatment prior to the assays to ensure that the signal is indeed derived from RNA-mediated interactions [for example, (Chu et al., 2015; Bell et al., 2018; Razin et al., 2020)].

Most of the RNA-interactome mapping methods rely on crosslinking to preserve the *in vivo* interactions followed by their detection *in vitro* (Table 1). To ensure that the detected interactions reflect *in vivo* situation, control experiments devoid of the crosslinking step are often included in different methodologies [for example, (Engreitz et al., 2013; Nguyen et al., 2016; Sharma et al., 2016)]. Still, such methods do have the disadvantage that their results may not fully reflect the *in vivo* situation. This problem can be remedied by the *in vivo* methods based on proximity labeling (Tables 1 and 2). However, such *in vivo* methods can only be used in cultured cells that are amenable to transfections (Table 2).

## Validation of the Interactome Mapping Methods and Novel Interactions Found by Them

Despite all the technical steps and controls developed and undertaken by the different methodologies to ensure specificity of their results, validation of the actual RNA interactions represents the basic key information required to understand the quality of the results provided by these techniques. Below, we review the different strategies used to validate the performance of the methods described above. One the most commonly used validations approaches is based on detection of relatively few “gold standard” interactions that have been extensively characterized and proven by years of studies that used multiple independent molecular biological, biochemical and genetic means; and typically involve highly abundant cellular RNAs. In terms of RNA-DNA interactome, such interactions include binding of the dosage compensation *roX1/2* or *XIST* lncRNAs to the respectively fly or mammalian X chromosomes (see above). Strong enrichment of the detected interaction sites for these lncRNAs on the X chromosomes compared to the autosomes was demonstrated by ChIRP (Chu et al., 2011), ChART (Simon et al., 2011), RAP (Engreitz et al., 2013), ChAR-seq (Bell et al., 2018), Red-C (Razin et al., 2020) and RD-SPRITE (Quinodoz et al., 2021) techniques. Furthermore, strong overlap between the ChIRP and CHART sites for *roX2* lncRNAs on the *Drosophila* X chromosome and those obtained independently by ChIP-seq with antibodies against protein components of the MSL complex provided additional proof for the specificity of these methodologies (Chu et al., 2011; Simon et al., 2011).

The “gold standard” inter-molecular RNA-RNA interactions are represented by binding of small nucleolar (sno) RNA to specific site on ribosomal (r) RNAs to mediate site-specific RNA modifications, known sites of interactions between the rRNAs and spliced mRNAs in the context of ribosome, and interactions of small nuclear (sn) RNAs components of spliceosome with each other or with pre-mRNAs, among others. For example, RIC-seq could detect specific enrichment of binding of U58A and U74 snoRNAs at their known binding sites on the 28S rRNA (Cai et al., 2020). Likewise, PARIS could show specific enrichment of binding of SNORD95 and U8 snoRNAs at the expected locations on 28S rRNA (Lu et al., 2016), and SPLASH could show the same for U42B and U80 snoRNAs on 18S and 28S rRNAs, respectively, (Aw et al., 2016). On the other hand, the LIGR-seq study reported detection of the expected interaction between the major U4-U6 and minor U4ATAC-U6ATAC spliceosomal snRNAs (Sharma et al., 2016). Interaction between U1 snRNA and MALAT1 lncRNA identified by RAP-RNA (Engreitz et al., 2014) has recently become another “gold standard” interaction used to validate, in part, performance of PARIS (Lu et al., 2016), RIC-seq (Cai et al., 2020), Proximity RNA-seq (Morf et al., 2019) and RD-SPRITE techniques (Quinodoz et al., 2021). Interestingly, RD-SPRITE detected the expected global enrichment of interactions of snRNAs with pre-mRNAs and rRNA with spliced mRNAs, but not snRNAs with mRNAs and rRNA with pre-mRNAs, which would be exactly expected for the authentic spliceosome and ribosome patterns and strongly supporting performance of this technique (Quinodoz et al., 2021).

Well-characterized protein components of spliceosome were also used to evaluate performance of *in vivo* RNA-centric proximity labeling method RPL (RNA proximity labelling) where U1 snRNA was targeted by dCas13 fused to APEX2 (Lin et al., 2021). Indeed, a number of spliceosomal proteins known to interact directly and indirectly with U1 were found (Lin et al., 2021). Likewise, detection of proteins previously found to interact with *XIST* lncRNA was used as a measure of validation for a different *in vivo* RNA-centric proximity labeling method CARPID (CRISPR-assisted RNA–protein interaction detection) also based on dCas13, but instead of APEX2, fused to a biotin ligase (Yi et al., 2020).

On the other hand, the RNA-centric and especially global methods have identified thousands and even millions of novel interactions: for example, Red-C found 44 M unique contacts between RNA and DNA in just one human cell type (Razin et al., 2020). These interactions involve novel interactions for classes of RNAs known to interact, for example novel snoRNAs binding sites on rRNAs, as well as interactions involving pairs of targets not known to interact previously. Detection of multiple novel RNA interactome events brings about questions of 1) their true existence in the cell as well as 2) biological relevance, answers to which we will attempt to summarize below. Different methods have been used to prove the authenticity of novel interactions based on biochemical, microscopical and genomic techniques. Biochemical approaches are typically based on isolation of one of the interacting partners followed by analysis of co-enrichment of another. For example, of the 122 novel snoRNA-rRNA interactions detected by SPLASH, the authors tested and could successfully validate 3 such interactions by pulldown of the RNA-RNA complexes with oligonucleotides against the rRNAs followed by reverse transcription quantitative PCR (RT-qPCR) detection of the co-enrichment of the snoRNAs (Aw et al., 2016). Furthermore, in the same study the authors could validate 12/13 out of ∼1,000 novel mRNA-mRNA interactions using this approach (Aw et al., 2016). In a different example, novel RNA-protein interactions between U1 snRNA and GTF2F2 and KPNB1 proteins detected by RPL (RNA proximity labelling) were confirmed using immunoprecipitations with antibodies against the two proteins followed by RT-qPCR detection of U1 RNA (Lin et al., 2021). Interestingly, one other novel RNA-protein interaction tested in that study was proven to be false positive (Lin et al., 2021), arguing for the necessity to validate the existence of the novel interactions detected by the high-throughput methods.

Microscopy-based approaches are based on co-localization of the two interacting partners *in vivo* using high-resolution microscopy. For example, RNA-RNA interactions between *Malat1* lncRNA and *Slc2a3* mRNA detected in mouse cells using MARIO method were confirmed *in vivo* by two-color single-molecule RNA fluorescence *in situ* hybridization (RNA-FISH) (Nguyen et al., 2016). Combined RNA-FISH and immunofluorescence analysis found co-localization of *PNCTR* lncRNA with hnRNPL and MCM5 proteins thus confirming these interactions detected by the HyPro-MS methodology (Yap et al., 2021). RNA/DNA co-FISH analysis could validate 4 out of 5 tested RNA-DNA interaction sites for the *NEAT1* lncRNA out of 1,251 total such sites found using CHART technology (West et al., 2014). A combination of both biochemical and microscopy-based approaches was used to support novel interaction between *XIST* lncRNA and TAF15 protein found by CARPID (Yi et al., 2020) (also see below).

However, the biochemical and microscopical validations are fairly labor intensive, therefore, as exemplified above, typically only a small number of observed interactions are confirmed using these methods. Typically, majority of the detected interactions are confirmed using other high-throughput methods. For example, RADICL-seq has mapped interactions between *Malat1* lncRNA and 10,000 genes in mouse genome (Bonetti et al., 2020). Of these, respectively 78% overlapped with sites previously detected by RAP technique in the same cell type, this confirming these sites and validating performance of RADICL-seq method in general (Bonetti et al., 2020).

## Estimating Distances Between the Interacting Molecules for Different Types of Methods

Distance between the interacting molecules is a very important parameter required for interpreting the data from the interactome mapping methods. Perhaps the clearest estimation of this parameter can be provided by the methods that are based on psoralen-derived or UV crosslinkers for RNA-RNA and RNA-protein interactions, respectively, that exclusively select for direct interactions between the molecules (Table 1, see above). The situation is far more complex for the methods that detect indirect, protein-mediated interactions or general co-localization (non-proximal interactions, Table 1). Perhaps the most specific distance measurements in this respect are available for the group of RNA-centric methods based on proximity labeling (Table 1). Typically, such methods quote a radius of ∼20 nm for the labeling zone (Rhee et al., 2013; Yap et al., 2021). Indeed, this estimate agrees quite well with the measurements conducted by the study of Lin et al. (2021) that used RPL (RNA proximity labelling, Figure 1A and Table 1) technique, to detect proteins interacting with U1 snRNA. Using molecular modelling based on 6 proteins whose interactions with U1 were detected by the method, the authors estimated that APEX2 enzyme can biotinylate proteins within 15 nm and the method detects targets within 25 nm of the RNA of interest (Lin et al., 2021), consistent with the above estimates.

The distances involved can only be mostly inferred for the other methods discussed here. Methods based on proximity ligations would be expected to enrich for proximal, nearby interactions that could either be direct or indirect (Table 1). However, the usage of bridging oligos that have typically tens of nucleotides in lengths [for example, 50 nt in RADICL-seq (Bonetti et al., 2020) and 37 nt in Red-C (Razin et al., 2020)] would potentially expand the distances that separate detectable molecules, however, the radiuses of detectable interactions have not been measured for any of the methods that map indirect interactions using proximity ligation.

Methods that rely on fragmentated crosslinked chromatin (Table 1) typically estimate the sizes of the resulting chromatin particles based on the sizes of the fragmented DNA. For example, the study by Morf et al. (2019) that developed Proximity RNA-seq has used a conversion coefficient of 0.01 nm per bp of DNA in chromatin fibers to estimate the resulting size distribution of particles from that of the fragmented DNA (Morf et al., 2019). To our knowledge, only a study from our group by Cao et al. (2021) that used the RAT technique (see below) directly measured sizes of fragmented crosslinked chromatin particles using flow sorting (Cao et al., 2021). Interestingly, we found that the size of the chromatin particles reached 300–500 nm, even though the fragmented DNA was below 500 bp (Cao et al., 2021). These results suggest that methods based on fragmented crosslinked chromatin can measure very distal interactions, presumable more distal than the methods that use proximity ligation (Cao et al., 2021). APEX-seq and APEX-MS methods were estimated to have resolution below 100 nm (Padron et al., 2019), however, it would likely depend on the dimensions of the subcellular locale to which APEX2 was targeted and precision of targeting.

## Biological Relevance of the Detected Interactions

The study by Bonetti et al. (2020) has reported that *Malat1* lncRNA could be found interacting with DNA sites in 14,158 mouse genes by either RADICL-seq, RAP or GRID-seq techniques. Of those, interactions with 5,883 (∼40%) genes could be found by all 3 techniques (Bonetti et al., 2020). However, such extensive interactome for that lncRNA contrasts with the fact that mice containing genetic knockouts of *Malat1* are healthy and have no obvious phenotypes as shown independently by 3 different groups (Eissmann et al., 2012; Nakagawa et al., 2012; Zhang et al., 2012). In a different example, ChIRP analysis has also found multiple—832—DNA interaction sites in human genome for lncRNA *HOTAIR* in just one cell type (Chu et al., 2011). However, the initial phenotypes observed for the *Hotair* knockout mice (Li et al., 2013) could not be reproduced, and, in fact, no phenotypes for *Hotair* knockout animals could be found in an independent follow-up study by another group (Amandio et al., 2016), reviewed in (Selleri et al., 2016). While the failure to obtain *in vivo* phenotypes does not necessarily invalidate the results of the RNA interactome mapping since, for example, *Malat1* could have *in vivo* function under certain conditions as reviewed in (Gao et al., 2020), it does however raise an issue of what fraction of the observed novel interactions have biological relevance. This issue is also exacerbated by the fact that different RNA interactome mapping methods do not always agree well. For example, the study by Bonetti et al. (2020) also compared detection of another non-coding RNA *Rn7sk* by ChIRP, RADICL-seq and GRID-seq and found that only 1,241 or ∼9% of the 13,970 interacting sites could be detected by all 3 methods.

Biological relevance of specific novel interactions has been tested using genetic means in several studies. For example, LIGR-seq detected multiple novel interactions between SNORD83B snoRNA and mRNAs (Sharma et al., 2016). Knockdown of the snoRNA led to up-regulation of the 3 out of 4 tested mRNAs where interactions were detected, while no change in expression was found for the 4 tested non-interacting control mRNAs (Sharma et al., 2016). The role of the above-mentioned *XIST*-*TAF15* interaction detected by CARPID in the X chromosome inactivation was confirmed by knockdown of TAF15 mRNA that has led to specific changes in gene expression on the inactive X chromosome (Yi et al., 2020). While these and other examples were highly informative to evaluate the biological roles of specific interactions, to our knowledge, the issue of the biological relevance of the newly discovered RNA interactomes have not yet been systematically addressed on a comprehensive, genome-wide level. Below, we will review the few reports that attempted to address this problem at genome-wide level.

The study by Yan et al. (2019) attempted an interesting analysis of overlap between RNA-DNA interactions found using iMARGI and locations of known gene fusions. Gene fusions are prominent in cancers where they often drive the malignant phenotypes. Interestingly, the study found that of the top 10 most significant RNA-DNA interactions found by iMARGI in normal cells, 5 correspond to known gene fusions found in cancers (Yan et al., 2019). Moreover, the authors detected statistically-significant overlap between known sites of cancers fusions and RNA-DNA interactions genome-wide (Yan et al., 2019). Specifically, the authors tested 6,253 inter- and 8,891 intra-chromosomal fusions and found strong statistically significant overlap with RNA-DNA interactions mapped using iMARGI for both types of fusions (Yan et al., 2019). Interestingly, the authors have also found a fusion transcript corresponding to genes involved in RNA-DNA interactions detected by iMARGI in the absence of the actual fusion on the DNA level (Yan et al., 2019). These results led the authors to propose the RNA-poise model to explain generation of gene fusions using 2 different mechanisms: 1) proximity of transcript product of gene 1 to gene and transcript products of gene 2 and 2) proximity of both transcripts and genes from both loci (Yan et al., 2019). In the case of any mechanisms, the proximity of RNA and DNA in the nucleus would have actual biological consequences as represented by the formation of gene and/or transcript fusions (Yan et al., 2019).

Three studies attempted to analyze genome-wide effects on the interactome of specific lncRNA transcripts following their genetic depletion. The study by Chu et al. (2017) has found thousands of chromatin interaction sites for telomeric repeat-containing RNAs (*TERRA*) using CHIRT technology. The authors have depleted *TERRA* transcripts using antisense oligonucleotides and found that expression levels of the 914 mouse genes containing binding sites of these non-coding transcripts were affected more significantly, either up- or down-regulated, than the 15,871 control genes without the binding sites (Chu et al., 2017). The study by Yap et al. (2021) attempted to compare the genome-wide effect of knockout of *NEAT1* lncRNA on the fate of transcripts found to interact with that lncRNA using the proximity labeling HyPro-seq methodology. They found that the transcripts that interacted with the lncRNA did have statistically-significant trend to be downregulated compared with the ones that did not, however, the authors did not comment on the actual numbers of transcripts used in the analysis (Yap et al., 2021).

The above-mentioned study by Cao et al. (2021) from our group used three approaches to study the function of very long intergenic non-coding (vlinc) RNAs: 1) correlation of expression levels between the vlincRNAs and all protein-coding mRNAs across multiple sample types, 2) CRISPR/Cas13 knockdown and 3) mapping vlincRNA-chromatin interactions by the above-mentioned RAT technique. VlincRNAs represent a widespread class of lncRNA transcripts with a minimum length of 50 kb (Kapranov et al., 2010). While members of this class were implicated in pluripotency and cancer (St Laurent et al., 2013), cellular survival following anticancer drug treatments (Xu et al., 2020), cell-cycle control (Heskett et al., 2020) and cellular senescence (Lazorthes et al., 2015), mechanisms of function and biological relevance of most of these transcripts remain unknown. The study by Cao et al. (2021) found that at the genome-wide level, a vlincRNA also had a stronger RNA-DNA interaction, indicating a closer proximity in the nucleus, with genes whose expression levels correlated with expression level of the vlincRNA. Furthermore, the study has shown that knock down of selected vlincRNAs using CRISPR/Cas13 affected the expression of the genes to which it was close in the nucleus and whose expression correlated with that of the vlincRNA (Cao et al., 2021). Altogether, these results argued that vlincRNAs can regulate expression of multiple other genes via a mechanism relying on proximity in the nucleus (Cao et al., 2021).

## Concluding Remarks

Mapping RNA interactome is a key to understanding molecular mechanisms of action of the RNA dark matter. In addition, deciphering interactome of both non-coding and protein-coding transcripts is also crucial to fully understand intricate details of the spatial organization, functioning and regulation of various subcellular compartments of the cell. It can also help us to better appreciate the molecular mechanisms responsible for generation of aberrant transcripts that are common in malignant states. Tremendous progress has been done in developing multiple methods to map RNA interactions at different levels. However, the abundance of data obtained with these technologies also naturally brings with it challenges in interpreting this information. Thus, comprehensive elucidation of the properties of mapped RNA interactions, such as for example, estimates of the distances between the interacting molecules mapped by the different methods and identification of biologically relevant RNA interactions, are critical for our appreciation of the complexities of the function and regulation of RNA dark matter, protein coding mRNAs and ultimately the cell.
